# Preoperative Prediction of Intracranial Meningioma Grade Using Conventional CT and MRI

**DOI:** 10.7759/cureus.21610

**Published:** 2022-01-25

**Authors:** Toshiyuki Amano, Akira Nakamizo, Hideki Murata, Yuichiro Miyamatsu, Fumihito Mugita, Koji Yamashita, Tomoyuki Noguchi, Shinji Nagata

**Affiliations:** 1 Neurosurgery, Kyushu Medical Center, Fukuoka, JPN; 2 Radiology, Kyushu Medical Center, Fukuoka, JPN

**Keywords:** relative growth rate, tumor doubling time, chronological changes, radiological features, preoperative diagnosis, intracranial high-grade meningioma

## Abstract

Objective

Preoperative diagnosis of tumor grade can assist in treatment-related decision-making for patients with intracranial meningioma. This study aimed to distinguish between high-grade and low-grade meningiomas using conventional CT and MRI.

Methodology

We retrospectively analyzed 173 consecutive patients with intracranial meningioma (149 low-grade and 24 high-grade tumors) who were treated surgically at the National Hospital Organization Kyushu Medical Center from 2008 to 2020. Clinical and radiological features, including tumor doubling time (Td) and relative growth rate (RGR), were compared between low-grade and high-grade meningiomas.

Results

Multivariate logistic regression analysis showed that symptomatic tumor (p=0.001), non-skull base location (p=0.006), irregular tumor shape (p=0.043), tumor heterogeneity (p=0.025), and peritumoral brain edema (p=0.003) were independent predictors of high-grade meningioma. In 53 patients who underwent surgery because of tumor progression, progression to symptoms (p=0.027), intratumoral heterogeneity (p<0.001), peritumoral brain edema (p=0.001), larger tumor volume (p=0.005), shorter Td (p<0.001), and higher RGR (P<0.001) were significantly associated with high-grade meningioma. Receiver operating characteristics (ROC) curve analysis showed that the optimal Td and annual RGR cut-off values to distinguish high-grade from low-grade meningioma were 460.5 days and 73.2%, respectively (100% sensitivity and 78.6% specificity).

Conclusion

Based on our findings, conventional CT and MRI are useful methods to predict meningioma grades before surgery. High-grade lesions are associated with non-skull base location, irregular tumor shape, intratumoral heterogeneity, and peritumoral brain edema. High-grade meningioma should be suspected in tumors that exhibit Td <460.5 days or annual RGR >73.2% or those that develop intratumoral heterogeneity or surrounding brain edema on surveillance imaging.

## Introduction

Intracranial meningioma is the most common primary brain tumor. These tumors typically have an indolent course due to their benign biological features. However, high-grade meningiomas have been associated with faster progression, higher recurrence, and lower survival despite multimodal treatment [1]. The World Health Organization (WHO) tumor grade is an effective predictor of tumor recurrence and overall survival. This classification system for brain tumors was first introduced in 1979 and most recently updated in 2016. Although several recent systematic reviews and meta-analyses have explored possible predictors of the WHO meningioma grade, high-quality evidence is still scarce [2-5]. Recent advances in novel imaging techniques such as machine learning-based radiomics analysis can improve the accuracy of radiological prediction of meningioma grade [6,7]. However, most imaging markers remain insufficient for routine clinical use because of poor tumor grade prediction accuracy [3]. Therefore, preoperative diagnosis of high-grade meningiomas remains a challenge.

The 2016 WHO classification incorporated cerebral invasion into the diagnostic criteria for WHO grade II meningioma. This modification has resulted in an increase in high-grade meningioma diagnosis, which is estimated to range between 17% and 31% [8,9]; based on the old WHO classification, the reported prevalence rates ranged from 5% to 10% [10]. Considering the cerebral invasion modification, radiological features of conventional CT and MRI may now be able to better predict meningioma grades.

In this study, we retrospectively analyzed 173 consecutive patients with histopathologically proven intracranial meningioma who underwent surgical resection at a single center. Clinical and radiological features were examined and compared between high-grade and low-grade tumors to assess the value of using preoperative CT and MRI to predict histopathological tumor grade.

## Materials and methods

This study was approved by the National Hospital Organization Kyushu Medical Center Research Ethics Board (#17C292). Informed consent was obtained from all patients. In our hospital, asymptomatic small intracranial meningiomas are treated conservatively and observed using repeated radiological assessment. When these tumors demonstrate chronological progression, surgical removal is considered. However, early surgical intervention is considered for some asymptomatic tumors based on their size or location before observing tumor growth. Decisions regarding surgical indications and the timing of surgery are made by the attending neurosurgeon.

Patient characteristics

This retrospective study examined the medical and radiological records of consecutive patients with histopathologically confirmed meningioma at the National Hospital Organization Kyushu Medical Center from 2008 to 2020. Patients with neurofibromatosis, spinal meningioma, or recurrent meningioma were excluded. A total of 173 patients were included for analysis (129 females and 44 males). The median age of the cohort was 65 years (range: 30-96). Age, gender, comorbidities, symptoms, tumor location, and histopathological grade were recorded. The histopathological grade was determined according to the 2016 WHO criteria. WHO grade I meningiomas were categorized as low-grade, and grade II or III meningiomas as high-grade. Presenting symptoms included cranial nerve palsy, motor weakness, memory disturbance, seizure, headache, ataxia, gait disturbance, and dysesthesia. Tumor location was categorized as skull base (including the olfactory groove, frontal base, tuberculum sellae, clinoidal region, sphenoid ridge, middle fossa, clivus, petrous ridge, tentorium cerebelli, jugular foramen, and foramen magnum) or non-skull base (including the convexity, parasagittal angle, falx cerebri, and within the ventricle).

Radiological findings

**Figure 1:**
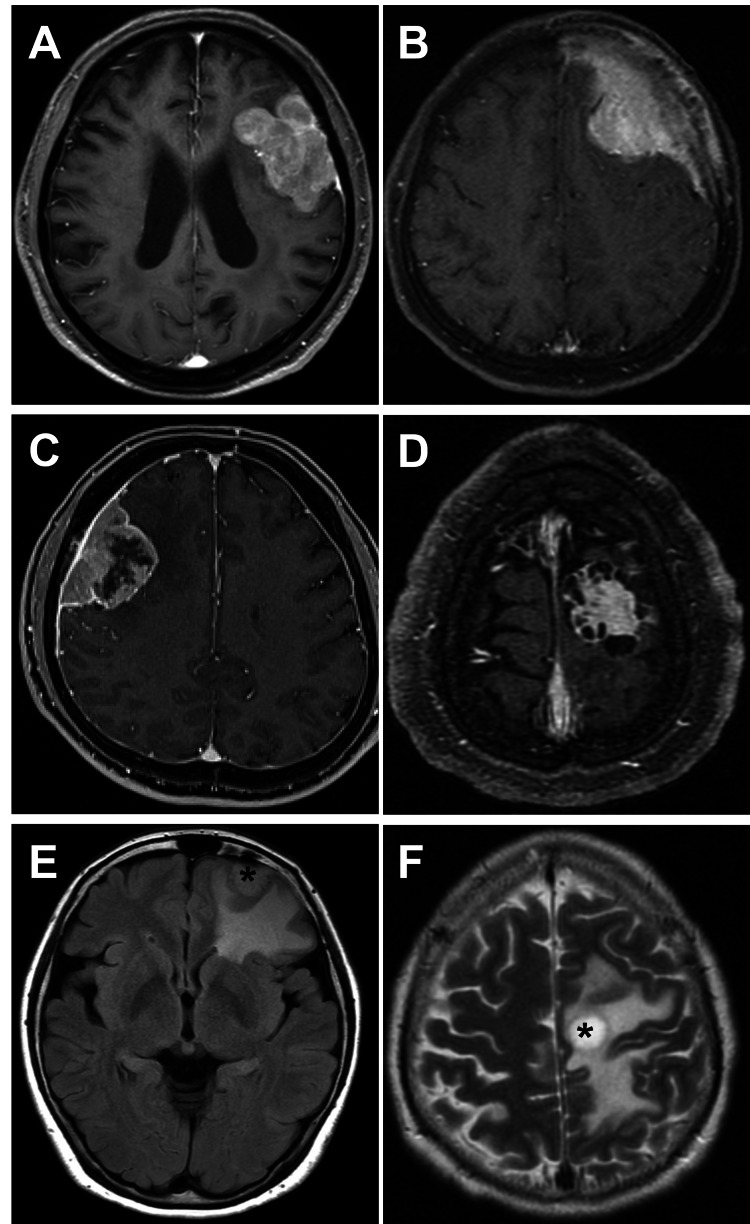
Representative MRI MRI showing representative axial images of irregular tumor shape, such as lobulated appearance (A) or mushrooming appearance (B), and heterogeneous tumor enhancement (C and D) on contrast-enhanced T1-weighted imaging and peritumoral brain edema on fluid-attenuated inversion recovery imaging (E) and T2-weighted imaging (F). The asterisk indicates tumor location MRI: magnetic resonance imaging

All intracranial meningiomas were confirmed by CT and MRI. Radiological images were interpreted by a senior neuroradiologist experienced in neuroimaging who was blinded to the histopathological results. Bone erosion, hyperostosis of the adjacent skull, and tumor calcification were assessed on CT. MRI was performed using a 1.5 T or 3 T scanner to obtain T1-weighted imaging (T1-WI), T2-weighted imaging (T2-WI), fluid-attenuated inversion recovery (FLAIR) imaging, and contrast-enhanced T1-WI. Tumor shape, intratumoral enhancement, and peritumoral brain edema were assessed on MRI. The irregular shape was defined as a lobulated (Figure 1A) or mushrooming appearance (Figure 1B). Intratumoral heterogeneity was defined as a heterogeneous enhancement on contrast-enhanced T1-WI (Figures 1C, 1D). Peritumoral brain edema was defined as a hyperintense signal change in the adjacent brain parenchyma on FLAIR or T2-WI (Figures 1E, 1F). Tumor size was measured as the maximum diameter on contrast-enhanced T1-WI. Tumor volume was calculated using the ellipsoid formula: (maximum length × maximum width × maximum height)/2 [2,11]. When serial clinical and imaging data were available, additional factors such as progressive symptoms, initial tumor volume, final tumor volume, time from diagnosis to surgery, tumor doubling time (Td), and relative growth rate (RGR) were also examined. Td was calculated using the following formula: T × log2/log (V_1_/V_0_), where T is the time between the initial and final volume, V_0_ is the initial tumor volume, and V_1_ is the final tumor volume [4,12]. RGR was calculated using the following formula: (2^(365/Td)^ −1) × 100 [4].

Statistical analyses

Statistical analyses were performed using the R software version 4.0.0 (R Foundation for Statistical Computing, Vienna, Austria) [13]. The chi-square test was used to compare categorical variables between the low-grade and high-grade groups; age, tumor volume, interval to surgery, Td, and RGR were compared using the Wilcoxon test. Variables significantly associated with high-grade meningioma in univariate analysis were used in multivariate logistic regression to assess independent predictors. The results of logistic regression are presented as odds ratios (ORs) with 95% confidence intervals (95% CI). The feasibility of Td and RGR as predictors of high-grade meningioma was determined using receiver operating characteristic (ROC) curve analysis. The point lying closest to the upper left corner of the ROC curve was defined as the optimal cutoff threshold value. A p-value <0.05 was considered statistically significant.

## Results

Clinical and radiological features associated with high-grade meningioma

Among the 173 meningioma patients, 149 (86.1%) harbored a low-grade tumor and 24 (13.9%) had a high-grade tumor. All high-grade tumors were WHO grade II. Patient characteristics are presented in Table 1. High-grade meningioma was significantly associated with older age (74 vs. 64 years; p=0.031), symptomatic tumor (79.2% vs. 38.3%; p<0.001), and non-skull base location (75.0% vs. 47.7%; p=0.023). However, gender (p=0.481), comorbidities (all p>0.1), and timing of surgical intervention (p=0.684) did not differ between the groups. In multivariate logistic regression analysis, symptomatic tumor (OR: 6.49; 95% CI: 2.33-21.34; p=0.001) and non-skull base location (OR: 4.39; 95% CI: 1.62-13.57; p=0.006) were independently associated with high-grade meningioma (Table 2).

**Table 1:** Clinical characteristics of 173 patients with intracranial meningioma IQR: interquartile range

Variables		Low-grade		High-grade	P-value
		n=149		n=24	
Age, years, median (IQR)		64 (56-72)		74 (57.5-82.25)	0.031
Gender, n (%)					0.481
	Female	113 (75.8)		16 (66.7)	
	Male	36 (24.2)		8 (33.3)	
Comorbidities, n (%)					
	Hypertension	51 (34.2)		11 (45.8)	0.384
	Dyslipidemia	29 (19.5)		7 (29.2)	0.415
	Cerebrovascular disease	18 (12.1)		2 (8.3)	0.850
	Diabetes mellitus	14 (9.4)		5 (20.8)	0.190
	Heart disease	12 (8.1)		3 (12.5)	0.743
	Cancer	9 (6.0)		2 (8.3)	1.000
	Liver dysfunction	3 (2.0)		2 (8.3)	0.290
	Chronic kidney disease	3 (2.0)		1 (4.2)	1.000
	Respiratory disease	2 (1.3)		0 (0)	1.000
Symptomatic, n (%)		57 (38.3)		19 (79.2)	<0.001
Tumor location, n (%)					0.023
	Skull base	78 (52.3)		6 (25.0)	
	Non-skull base	71 (47.7)		18 (75.0)	
Timing of surgical intervention, n (%)					0.684
	Surgery at initial diagnosis	102 (68.5)		18 (75.0)	
	Surgery after progression	47 (31.5)		6 (25.0)	

**Table 2:** Multivariate logistic regression analysis of associations between variables and high-grade meningioma in 173 patients OR: odds ratio; CI: confidence interval

Variables	Multivariate analysis		
	OR	95% CI	P-value
Age	1.04	1.00-1.08	0.074
Symptomatic tumor	6.49	2.33-21.34	0.001
Non-skull base tumor	4.39	1.62-13.57	0.006

Radiological findings are presented in Table 3. Bone erosion or hyperostosis in the adjacent skull was present in 139 patients (34 patients harbored tumors of the falx cerebri, tentorium cerebelli, or within the ventricle that were not adjacent to the skull). There were no significant differences in terms of hyperostosis (p=0.214) or calcification (p=0.155) between the low-grade and high-grade meningioma groups. Frequencies of bone erosion in the adjacent skull (35.0% vs. 10.9%; p=0.013), irregular tumor shape (45.8% vs. 7.4%; p<0.001), tumor heterogeneity (41.7% vs. 5.4%; p<0.001), and peritumoral edema (91.7% vs. 34.2%; p<0.001) were significantly higher in the high-grade meningioma group. Preoperative tumor volume was significantly higher in the high-grade meningioma group (30.6 cm^3^ vs. 8.3 cm^3^; p<0.001). In multivariate logistic regression analysis (Table 4), the following variables were independently associated with high-grade meningioma: irregular tumor shape (OR: 4.04; 95% CI: 1.04-16.11; p=0.043), intratumoral heterogeneity (OR: 5.62; 95% CI: 1.28-27.57, p=0.025), and peritumoral brain edema (OR: 13.35; 95% CI: 2.85-99.35; p=0.003).

**Table 3:** Radiological findings of 173 patients with intracranial meningioma IQR: interquartile range

Variables	Low-grade		High-grade	P-value
	n=149		n=24	
Bone erosion, n (%)	13/119 (10.9)		7/20 (35.0)	0.013
Hyperostosis, n (%)	14/119 (11.8)		5/20 (25.0)	0.214
Calcification, n (%)	57/149 (38.3)		5/24 (20.8)	0.155
Irregular tumor shape, n (%)	11/149 (7.4)		11/24 (45.8)	<0.001
Intratumoral heterogeneity, n (%)	8/149 (5.4)		10/24 (41.7)	<0.001
Peritumoral brain edema, n (%)	51/149 (34.2)		22/24 (91.7)	<0.001
Tumor volume, cm^3^, median (IQR)	8.3 (3.7-19.7)		30.6 (16.7-50.8)	<0.001

**Table 4:** Multivariate logistic regression analysis of associations between variables and high-grade meningioma in 173 patients OR: odds ratio; CI: confidence interval

Variables	Multivariate analysis		
	OR	95% CI	P-value
Bone erosion	1.92	0.42-8.29	0.383
Irregular tumor shape	4.04	1.04-16.11	0.043
Intratumoral heterogeneity	5.62	1.28-27.57	0.025
Peritumoral brain edema	13.35	2.85-99.35	0.003
Tumor volume	0.99	0.97-1.01	0.306

Chronological changes associated with high-grade meningioma

**Figure 2:**
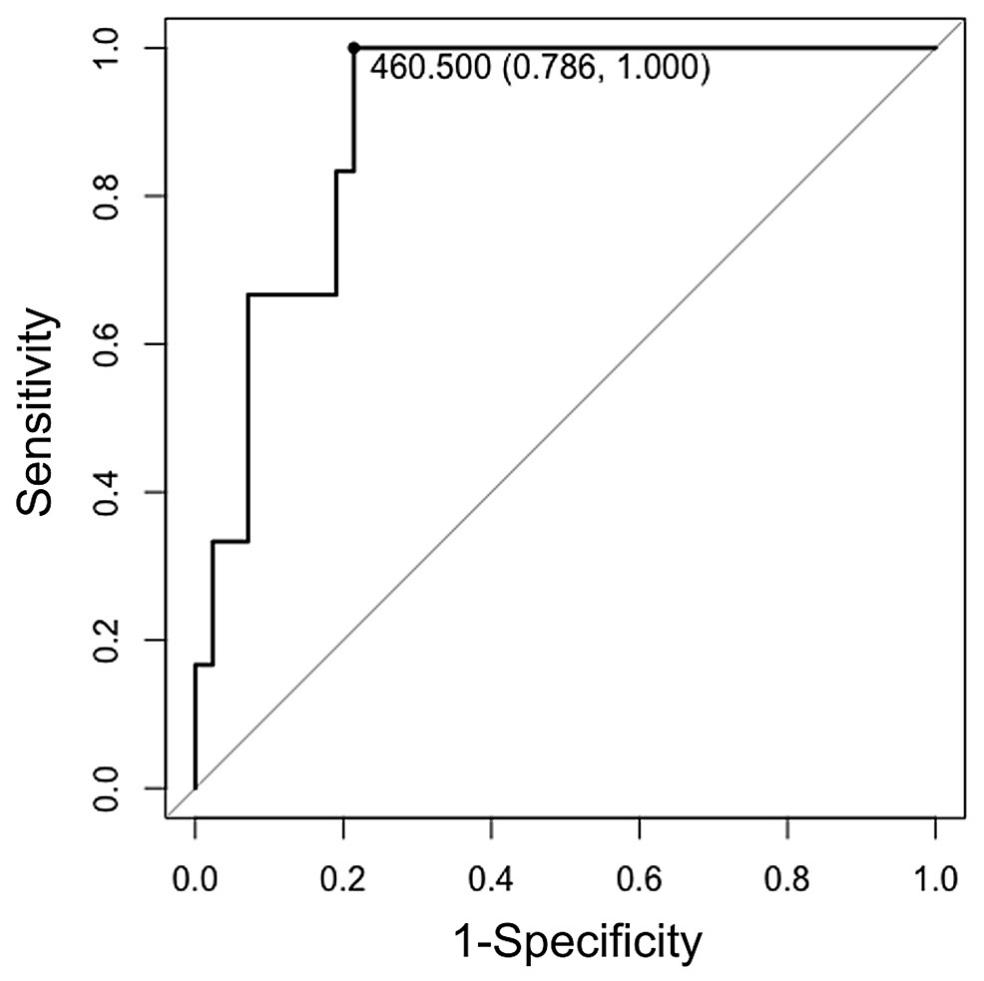
The receiver operating characteristic (ROC) curve of tumor doubling time (Td) in 53 progressive tumors The receiver operating characteristics (ROC) curve of tumor doubling time (Td) in progressive tumors to differentiate between high-grade and low-grade meningioma. The area under the curve was 0.90. The optimal cut-off value was 460.5 days. The specificity and sensitivity were 78.6% and 100%, respectively

**Figure 3:**
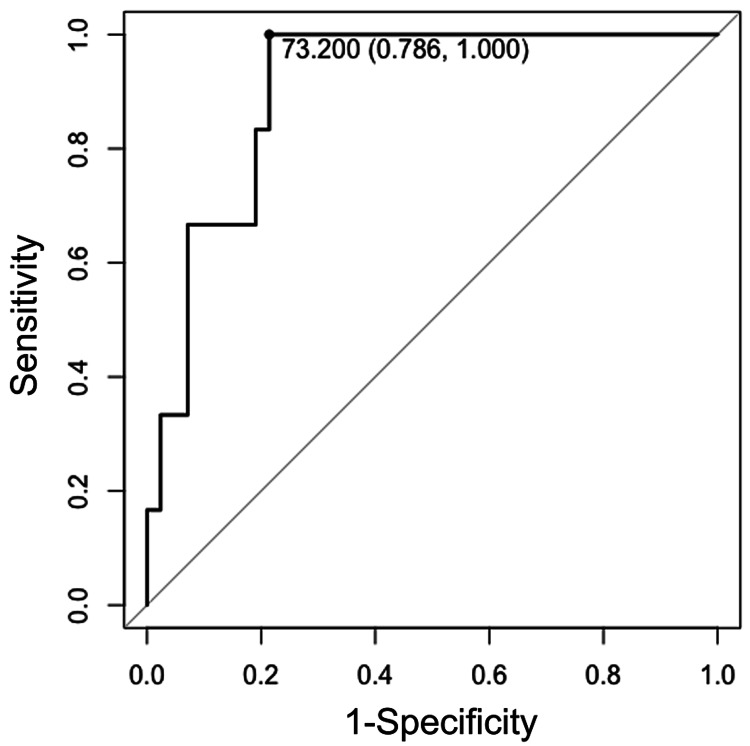
The receiver operating characteristic (ROC) curve of relative growth rate (RGR) in 53 progressive tumors The receiver operating characteristics (ROC) curve of relative growth rate (RGR) in progressive tumors to differentiate between high-grade and low-grade meningioma. The area under the curve was 0.90. The optimal cut-off value was 73.2% per year. The specificity and sensitivity were 78.6% and 100%, respectively

As shown in Table 1, 53 patients (47 with low-grade and six with high-grade meningioma) underwent a surgical intervention because of tumor progression. Among these patients, we investigated chronological changes in clinical and radiological characteristics, which are shown in Table 5. High-grade meningioma was significantly associated with progression to symptoms (66.7% vs. 17.0%; p=0.027), acquisition of intratumoral heterogeneity (66.7% vs. 2.1%; p<0.001) and peritumoral brain edema (100% vs. 23.4%; p=0.001), larger final tumor volume (28.7 cm^3^ vs. 5.9 cm^3^; p=0.005), shorter Td (304 days vs. 1038 days; p<0.001), and higher RGR (133% vs. 28%; p<0.001). However, there were no differences in terms of age (p=0.066), gender (p=0.549), acquisition of irregular tumor shape (p=0.534), initial tumor volume (p=0.066), and time from diagnosis to surgery (p=0.218) between the groups. ROC curve analysis showed that Td could distinguish between high- and low-grade meningiomas with a sensitivity of 100% and specificity of 78.6% using a cut-off of 460.5 days [area under the curve (AUC): 0.90; Figure 2]. RGR could distinguish between the two with a sensitivity of 100% and specificity of 78.6% using a cut-off of 73.2% per year (AUC: 0.90; Figure 3).

**Table 5:** Clinical and radiological features of 53 patients with progressive intracranial meningioma IQR: interquartile range

Variables		Low-grade		High-grade		P-value
		n=47		n=6		
Age, years, median (IQR)		68 (60-73)		82 (78.75-83.75)		0.066
Gender, n (%)						0.549
	Female	38 (80.9)		6 (100)		
	Male	9 (19.1)		0 (0)		
Progression to symptoms, n (%)		8 (17.0)		4 (66.7)		0.027
Acquired radiological features, n (%)						
	Irregular tumor shape	1 (2.1)		1 (16.7)		0.534
	Intratumoral heterogeneity	1 (2.1)		4 (66.7)		<0.001
	Peritumoral brain edema	11 (23.4)		6 (100)		0.001
Initial tumor volume, cm^3^, median (IQR)		1.5 (0.7-3.4)		4.0 (2.9-7.3)		0.066
Final tumor volume, cm^3^, median (IQR)		5.9 (2.6-9.8)		28.7 (19.8-32.0)		0.005
Time from diagnosis to surgery, days, median (IQR)		1060 (625-1605)		638 (427-977)		0.218
			Tumor doubling time, days, median (IQR)		1038 (504-1614)			304 (174-424)		<0.001
Relative growth rate, %, median (IQR)		28 (17-65)		133 (84-415)		<0.001

## Discussion

Clinical and radiological features associated with high-grade meningioma

In the present study, we attempted to determine whether preoperative CT and MRI can distinguish WHO high-grade intracranial meningioma from the low-grade variant. Although previous studies have also explored possible predictors of meningioma grade or subtype based on clinical characteristics or radiological features, the results have been conflicting. For example, the presence of peritumoral brain edema has been reported as a predictor of higher tumor grade in some studies [1,14] but not in others [15,16]. Therefore, preoperative diagnosis of high-grade meningiomas remains both controversial and challenging, and hence further data and analysis are still needed. In agreement with other studies, our research showed that symptomatic tumor [17], non-skull base location [14,17], irregular tumor shape [1,14,16], intratumoral heterogeneity [1,6,16], and the presence of peritumoral brain edema appear to be independent predictors of high-grade meningioma. The fact that clinical and radiologic findings can change over time must be considered when evaluating meningioma patients. The distinctive radiological features mentioned above may not be detected in newly arising meningiomas, even high-grade ones; rather, they may be gradually acquired as the tumor progresses. Therefore, although these radiological features can be associated with high-grade meningioma, their absence does not always indicate that the tumor is benign. Taken together, earlier surgical intervention with more radical resection should be considered in cases of non-skull base location, irregular tumor shape, intratumoral heterogeneity, and peritumoral brain edema, even in patients with an incidental tumor.

In contrast with previous studies, older age, male gender, comorbidities, bone erosion, absence of calcification, and tumor volume were not independently associated with high-grade meningioma in our study [7,16-18]. These conflicting results are likely due to variations in study design and population between studies. However, all studies, including ours, were conducted among patients with histopathologically confirmed meningioma; patients with an apparent meningioma followed conservatively or treated with radiosurgery without biopsy were excluded. In patients with an asymptomatic small tumor, conservative treatment or radiotherapy is generally preferred for older patients in poor condition, while surgical intervention may be preferred for younger healthy patients. Treatment decision-making for asymptomatic meningiomas is based on patient characteristics such as age, symptoms, and comorbidities as well as tumor characteristics such as location and size; patient preference may also be a factor [19]. Sample selection bias may have been present in several studies, making comparison difficult. To address this issue, standardized criteria should be established and used in future studies.

Chronological changes in progressive meningioma

With the widespread use of CT and MRI and advances in imaging techniques, incidental asymptomatic intracranial meningiomas are frequently detected these days. Nakasu and Nakasu, in a systematic review and meta-analysis of meningioma natural history, have shown that approximately 30% of incidental meningioma do not grow further [4]. When a meningioma has no distinct radiological high-grade features, conservative management is a reasonable option for asymptomatic small tumors. However, approximately 70% of intracranial meningiomas show chronological progression. Earlier establishment of tumor grade in progressive tumors can assist in treatment decision-making. When serial imaging data are available, chronological changes in tumor characteristics can be analyzed. We explored potential factors that distinguish between high- and low-grade lesions in progressive meningiomas. Some progressive meningiomas did not initially have the distinctive clinical and radiological features mentioned above but acquired them during observation. Among these, progression to symptoms, intratumoral heterogeneity, and peritumoral brain edema were significantly associated with high-grade meningioma; however, irregular tumor shape was not. This suggests that intratumoral heterogeneity and peritumoral brain edema are likely to be present in progressive high-grade meningioma. Development of these changes on imaging should be a focus of observation and raise suspicion for a high-grade tumor when identified.

Larger final tumor volume, shorter Td, and higher RGR were significantly associated with high-grade meningioma in our study. Although previous studies have also suggested an association between shorter Td and high-grade tumors [20-22], these studies used the old WHO meningioma classification, were limited by small sample size, did not conduct statistical analyses, and some of them included patients with unknown histology. To our knowledge, our study is the first to demonstrate that Td is significantly associated with meningioma grade and can be a reliable predictor of high-grade meningioma. Our ROC curve analysis showed a high AUC value (0.90) for both Td and RGR, indicating excellent performance at distinguishing high-grade from low-grade. The optimal Td cut-off value was 460.5 days, suggesting that tumor volume doubling within 15.2 months likely indicates high-grade meningioma. The optimal RGR cut-off value was 73.2% per year. This growth is equivalent to an estimated 20% increase in maximum length, width, and height. Such growth measured in one year on radiological surveillance imaging probably indicates high-grade meningioma. Taken together, Td and RGR can be possible predictors of high-grade meningioma even in tumors without other concerning radiological findings.

Limitations

This study has several limitations. Firstly, the retrospective single-center design may have introduced biases that affect the generalizability of our findings. For example, the surgical indications and timing in each patient were dependent on the individual treating neurosurgeon, and selection bias may have been present. Hence, future studies using established standardized surgical criteria are required. Secondly, the sample size was relatively small, which may have contributed to the lack of significance in some analyses. Because de novo intracranial high-grade meningioma is relatively rare, future multicenter studies are necessary to confirm our findings.

## Conclusions

In the present study, we attempted to correlate clinical and radiological features of intracranial meningioma with histopathological grading according to the revised 2016 WHO classification. Based on our findings, symptomatic tumor, non-skull base location, irregular tumor shape, intratumoral heterogeneity, and peritumoral brain edema were independent predictors of high-grade meningioma. When these distinctive clinical and radiological features are observed in patients with suspected meningioma, earlier surgical intervention with more radical resection should be considered.

In progressive meningiomas, those that developed intratumoral heterogeneity and peritumoral brain edema were likely to be high-grade. Furthermore, shorter Td and higher RGR were significantly associated with tumor grade, suggesting that these parameters may be predictors of high-grade meningioma. When evaluating chronological changes in meningioma, the presence of intratumoral heterogeneity, peritumoral brain edema, Td <460.5 days, and annual RGR >73.2% should raise suspicion for a high-grade tumor.
